# A theoretical comparison of position estimation methods for determining the interaction position of gamma rays in monolithic scintillators

**DOI:** 10.1186/2197-7364-2-S1-A36

**Published:** 2015-05-18

**Authors:** Matteo Galasso, Cristian Borrazzo, Andrea Fabbri

**Affiliations:** Department of Molecular Medicine, University of Rome "La Sapienza", Rome, Italy; INFN Sezione Roma III, Rome, Italy

We report the development of a mathematical model, based on the estimation theory, for the performance analysis of different gamma-ray interaction position estimation methods applied to gamma cameras based on monolithic scintillators. The gamma camera performance is expressed in terms of bias and spatial resolution. We propose a method to investigate performances of various detection system configurations, used in recent papers, when different image reconstruction algorithms are used. The results are obtained for the whole field of view, with a computational time five order of magnitude lower than a Monte Carlo simulation. We developed a mathematical model for four estimation methods: the classical Center of Gravity method (Anger Logic), an enhanced Center of Gravity method, a Mean Square Error fitting method and the Maximum Likelihood method. Moreover, those models can be used to investigate edge effects and performance of a detector with an arbitrary pattern of active elements and dead areas, in order to study systems with more multianode PMTs or SiPM array modules. The bias and spatial resolution results obtained with those mathematical model were compared with the ones obtained from Monte Carlo simulations, made with GEANT4, showing a very good agreement for all the methods.

